# Inhibitory Effects of Pinostilbene Hydrate on Melanogenesis in B16F10 Melanoma Cells via ERK and p38 Signaling Pathways

**DOI:** 10.3390/ijms21134732

**Published:** 2020-07-02

**Authors:** You Chul Chung, Chang-Gu Hyun

**Affiliations:** Jeju Inside Agency & Cosmetic Science Center, Department of Chemistry and Cosmetics, Jeju National University, Jeju 63243, Korea; jyc8385@hanmail.net

**Keywords:** B16F10 melanoma cell, p38, ERK, whitening, pinostilbene hydrate (PH), anti-melanogenesis

## Abstract

Melanin protects our skin from harmful ultraviolet (UV) radiation. However, when produced in excess, it can cause hyperpigmentation disorders, such as melanoma, freckles, lentigo, and blotches. In this study, we investigated the effects of pinostilbene hydrate (PH) on melanogenesis. We also examined the underlying mechanisms of PH on melanin production in B16F10 cells. Our findings indicated that PH significantly inhibits melanin content and cellular tyrosinase activity in cells without causing cytotoxicity. In addition, Western blot analysis showed that PH downregulated the protein levels of microphthalmia-associated transcription factor (MITF), tyrosinase, and other melanogenic enzymes, such as tyrosinase-related protein-1 (TRP-1) and tyrosinase-related protein-2 (TRP-2). Although PH activated the phosphorylation of extracellular signal-regulated kinase (ERK), it inhibited p38 mitogen-activated protein kinases (p38). Furthermore, the inhibition of tyrosinase activity by PH was attenuated by treatment with PD98059 (a specific ERK inhibitor). Additionally, p-AKT was upregulated by PH treatment. Finally, the inhibitory effects of PH on melanin content and tyrosinase activity were confirmed in normal human melanocytes. These results suggest PH downregulates melanogenesis via the inhibition of MITF expression, followed by the MAPKase signaling pathways. Thus, PH may be used to treat or prevent hyperpigmentation disorders and in functional cosmetic agents for skin whitening.

## 1. Introduction

Melanin is composed of pheo- and eumelanin. It is synthesized by specialized organelles, melanosomes, in melanocytes in the skin after exposure to stimulating factors, including UV light and stress. Melanocytes are located in the epidermal‒dermal junction and transfer melanosomes to the surrounding keratinocytes. The primary role of melanin, in particular eumelanin, is to determine the coloration of human skin, eyes, and hair. In addition, it protects against photo-damage caused by ultraviolet radiation by scavenging free radicals or dispersing UV light [1,2,3,4,5,6]. However, the excessive expression of melanin can result in the development of hyperpigmentation disorders, including melanoma, freckles, lentigo, and blotches [7,8].

Melanogenesis is related to a complex signaling process that occurs within melanocytes and involves melanogenic enzymes, such as tyrosinase and tyrosinase-related protein (TRP)-1 and -2. Tyrosinase is a key enzyme in the process of melanogenesis and catalyzes the oxidation of l-tyrosine and l-3,4-dihydroxyphenylalanine (L-DOPA) into DOPAquinone. DOPAquinone undergoes intramolecular cyclization to form cycloDOPA, which is further oxidized into DOPA chrome. DOPA chrome is then transformed into 5,6-dihydroxyindole-2-carboxylic acid (DHICA) and 5,6-dihydroxyindole (DHI) by dopachrome tautomerase (TRP-2) and tyrosinase. Finally, DHICA is oxidized by TRP-1 or tyrosinase to produce eumelanin [9,10,11,12].

Recent studies have indicated that microphthalmia-associated transcription factor (MITF) expression is strongly related to melanogenic enzyme expression, which contributes to melanogenesis in B16F10 cells [13,14,15]. That is, when the skin is stimulated by ultraviolet radiation (UVR), the production of intracellular α-melanocyte-stimulating hormone (α-MSH) increases, which binds to melanocortin receptor 1 (MC1R) and activates adenylate cyclase (AC) to increase cAMP levels. Then, increased cAMP enhances MITF transcription by increasing protein kinase A (PKA) and cAMP response element-binding protein (CREB) phosphorylation [16,17].

The mitogen-activated protein kinase (MAPK) family, comprised of extracellular signal-regulated kinase (ERK), c-Jun N-terminal kinase (JNK), and p38, has been implicated in melanogenesis through the regulation of MITF expression [18,19,20]. An increased phosphorylation of ERK decreases the transcription of tyrosinase activity and MITF, which inhibits melanin synthesis [21]. In addition, decreased JNK and p38 phosphorylation reduces the expression of MITF, leading to anti-melanogenesis [22,23]. Moreover, it has been reported that the activation of the protein kinase B (AKT) signaling pathway is related to increased MITF degradation and decreased melanogenic enzyme expression, thereby inhibiting melanogenesis [24,25]. Therefore, according to recent studies, the MAPK/AKT signaling pathway is considered a strategic target for the regulation of melanogenesis.

Resveratrol (3,5,4′-trihydroxy-trans-stilbene) is a polyphenolic compound and a phytoalexin that is produced by several plants when wounded or under attack by bacteria or fungi. Resveratrol is found in berries, peanuts, grapes, and it has antioxidative, neuroprotective, and cardioprotective properties [26,27,28,29,30,31]. Many researchers have studied this compound for potential applications in human health. However, resveratrol is unstable in the environment; it is very sensitive to UVR, air, and strong pH and is easily deformed. Thus, many resveratrol derivatives have been studied, and studies have found that methylated resveratrol derivatives have a good bioavailability and bioactivity [32,33,34]. One of the methylated resveratrol derivatives, pinostilbene hydrate (3,4′-dihydroxy-5-methoxystilbene), has been demonstrated to exert strong neuroprotective activity, as well as have anti-cancer effects on human colon cancer cells and anti-metastatic effects on human oral squamous cell carcinoma (OSCC) cells [34,35,36,37]. In addition, glycosylated pinostilbene hydrate has a radical scavenging activity and an inhibitory effect on tyrosinase [38]. However, the anti-melanogenic effects of pinostilbene hydrate in B16F10 melanoma cells has not yet been investigated. Therefore, in this study, we investigated the anti-melanogenic effects of pinostilbene hydrate (PH) on B16F10 cells to elucidate the underlying inhibitory mechanism.

## 2. Results

### 2.1. Effects of PH on the Viability of B16F10 Melanoma Cells

**Figure 1:**
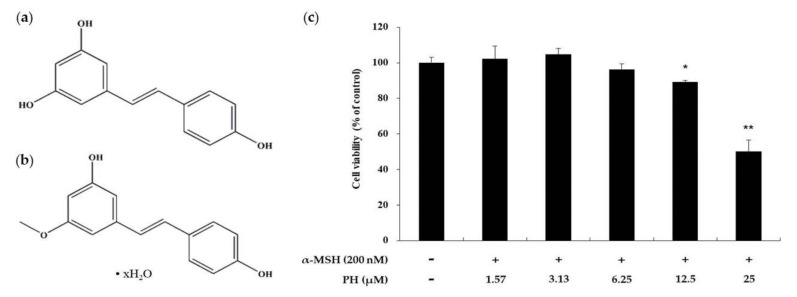
Effects of pinostilbene hydrate (PH) on the viability of B16F10 cells. Chemical structure of (**a**) resveratrol and (**b**) pinostilbene hydrate. (**c**) The cells were treated with various concentrations of PH for 72 h. Cell viability is expressed as percentages relative to untreated cells. The data are presented as the mean ± standard deviation (SD) of at least three independent experiments. * *p* < 0.05, ** *p* < 0.01.

Cell viability was assessed using a 3-(4,5-dimethylthiazol-2-yl)-2,5-diphenyltetrazolium bromide (MTT) assay. To determine the treatment range without PH cytotoxicity against B16F10 cells, the cells were treated with various concentrations of PH (1.57–25 µM) for 72 h. The results showed that the viability of the cells decreased by 65% after treatment with 25 µM PH. Otherwise, there was no cytotoxic effect on cell proliferation at the indicated concentrations (1.57, 3.13, 6.25, and 12.5 µM) compared with the untreated control cells (Figure 1). PH at 1.57, 3.13, 6.25, and 12.5 µM was used for subsequent experiments with B16F10 cells.

### 2.2. Effects of PH on Melanin Content and Tyrosinase Activity

**Figure 2:**
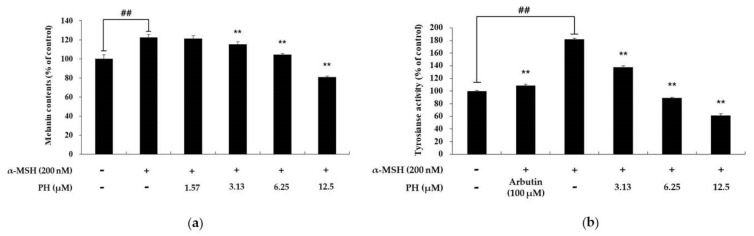
Effects of PH on melanin production and tyrosinase activity in B16F10 melanoma cells. Cells were treated with PH at the indicated concentrations for 72 h. α-MSH (200 nM) and arbutin (100 μM) were used as a negative control and positive control, respectively. (**a**) The melanin content and (**b**) tyrosinase activity are expressed as percentages relative to untreated cells. All experiments were carried out in triplicate, and the results are expressed as mean ± SD. ^##^ *p* < 0.01 in comparison with the untreated cell group and **p < 0.01. compared with the α-MSH-treated group.

To confirm the effects of PH on melanin synthesis and tyrosinase activity, cells were treated with the indicated concentrations of PH, followed by tests to determine the melanin content and tyrosinase activity. α-MSH (200 nM) and arbutin (100 μM) were used as negative and positive controls, respectively. As shown in Figure 2a, the melanin content of α-MSH-treated cells increased by 21% compared with the untreated control cells. In contrast, the highest concentration of PH (12.5 µM) treatment significantly decreased melanin synthesis by 34%, compared with α-MSH-treated cells. To determine the role of tyrosinase activity in the inhibitory effects of PH on melanin synthesis, we performed a tyrosinase activity test. As shown in Figure 2b, the tyrosinase activity of the negative control group increased by approximately 78% compared with the unstimulated cells. The tyrosinase activity of the 3.13, 6.25, and 12.5 µM PH-treated groups was found to decrease by 22%, 52%, and 63%, respectively, compared with the negative control group. The tyrosinase activity in the positive control group decreased by 38% compared with the negative control group. These results indicated that the inhibitory effects of PH on melanin synthesis is strongly related to the inhibition of tyrosinase activity.

### 2.3. Effects of PH on Melanogenic Enzymes and MITF Expression in B16F10 Cells

**Figure 3:**
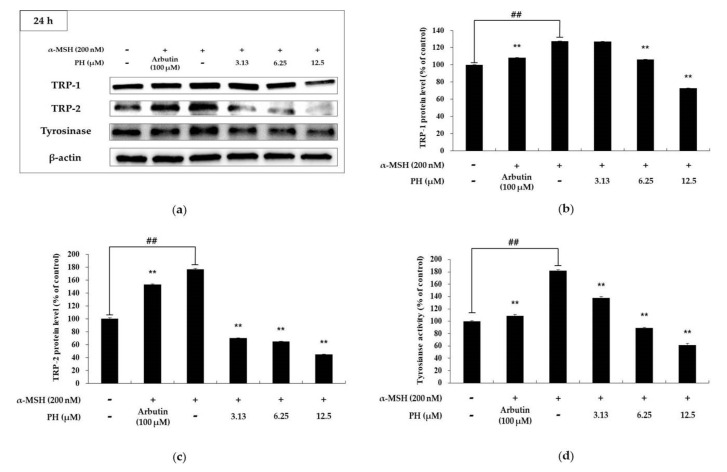
Effects of PH on the protein expression levels of TRP-1, TRP-2, and tyrosinase in B16F10 cells. Cells were treated with the indicated concentrations of PH for 24 h. α-MSH (200 nM) and arbutin (100 μM) were used as a negative and positive control, respectively. Protein levels were examined by Western blotting. (**a**) Western blotting and protein levels of (**b**) TRP-1, (**c**) TRP-2, and (**d**) tyrosinase. Results are expressed as percentages of untreated cells. All experiments were carried out in triplicate, and the results are expressed as the mean ± SD. ^##^ *p* < 0.01 in comparison with the untreated cell group and ** *p* < 0.01 compared with the α-MSH-treated group.

**Figure 4:**
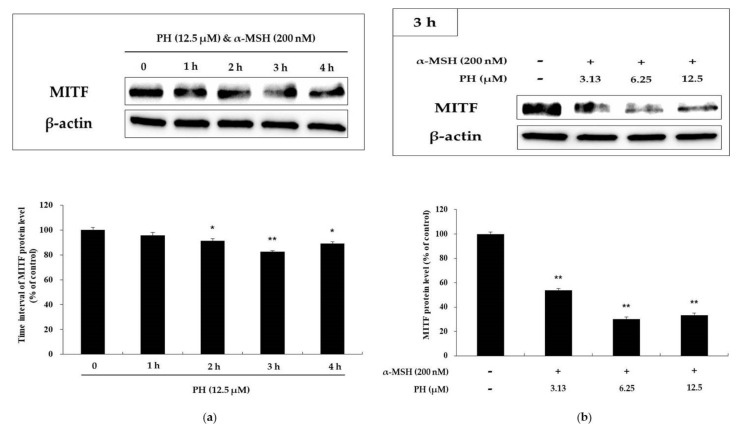
Effects of PH on MITF expression in B16F10 cells. (**a**) Cells were treated with 12.5 µM PH at different time intervals. (**b**) Cells were treated with the indicated concentrations of PH for 3 h. Protein levels were examined by Western blotting. Results are expressed as percentages of the untreated cells. All experiments were carried out in triplicate, and the results are expressed as the mean ± SD. * *p* < 0.05, ** *p* < 0.01.

Melanogenic enzymes, such as tyrosinase and TRP-1 and -2, play important roles in the process of melanogenesis. MITF, a transcriptional factor, regulates melanogenic enzymes and melanogenesis. To determine the expression of these proteins, we performed a Western blotting assay [13,14,15]. As shown in Figure 3, the expression of melanogenic enzymes in B16F10 cells was found to be significantly decreased in a concentration-dependent manner, compared with α-MSH-treated cells. In particular, the highest concentration PH treatment groups showed significantly reduced protein expression relative to the untreated control cells. To understand the transcriptional regulation of melanogenic enzyme inhibition, we evaluated the effects of PH on MITF expression. As shown in Figure 4a, the time interval results indicated that PH treatment for 3 h reduced MITF expression the most. As a result of processing PH for 3 h in a subsequent experiment, it was confirmed that MITF expression was decreased in a concentration-dependent manner (Figure 4b). These results demonstrated that melanogenic enzyme expression was reduced by the attenuation of MITF expression.

### 2.4. Effects of PH on the MAPK Signaling Pathway in B16F10 Cells

**Figure 5:**
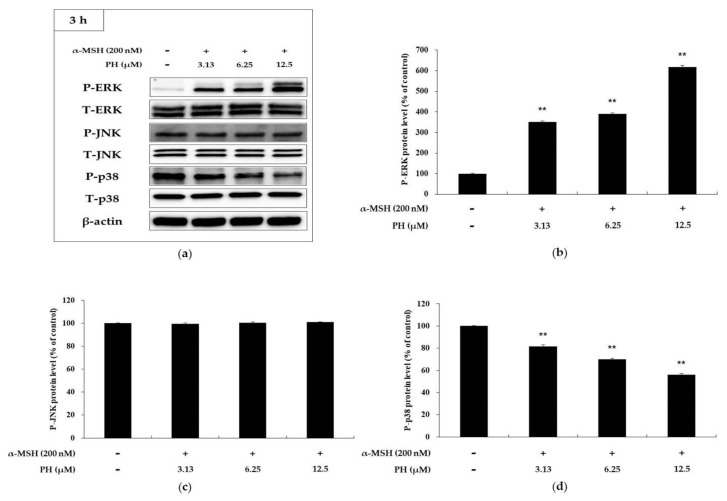
Effects of PH on phosphorylation of ERK, p38, and JNK. B16F10 cells treated with PH at the indicated concentrations for 3 h. (**a**) Western blotting results and protein levels of (**b**) p-ERK, (**c**) p-JNK, and (**d**) p-p38. Results are expressed as percentages of the untreated cells. All experiments were carried out in triplicate, and the results are expressed as the mean ± SD. ** *p* < 0.01. P: phosphorylated, T: total.

**Figure 6:**
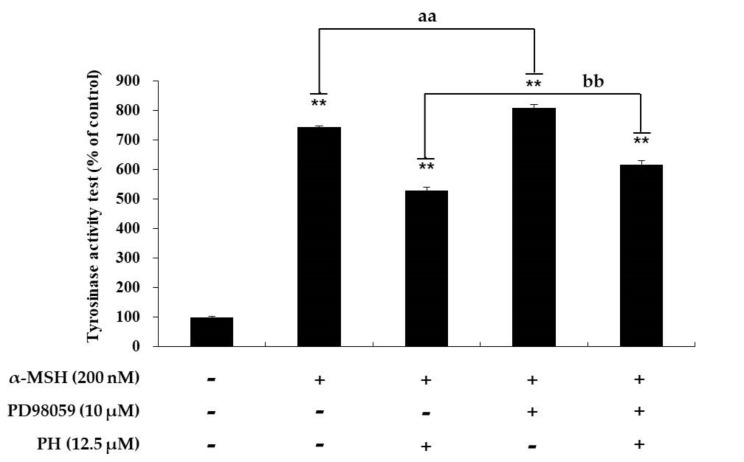
Effects of PD98059, an ERK inhibitor, on tyrosinase activity in B16F10 cells. Cells were treated with α-MSH (200 nM) or PD98059 (10 μM) alone or in combination with or without PH at a single concentration of 12.5 µM. Results are expressed as percentages of the untreated cells. All experiments were carried out in triplicate, and the results are expressed as the mean ± SD. ** *p* < 0.01, ^aa^ *p* < 0.01, ^bb^ *p* < 0.01.

To confirm that PH inhibits MITF expression via the MAPK signaling pathway, MAPK phosphorylation was investigated. As shown in Figure 5, PH significantly increased ERK phosphorylation and decreased p38 in a concentration-dependent manner. However, it did not affect JNK phosphorylation. To determine whether an increase in ERK phosphorylation is involved in inhibiting tyrosinase activity, a specific ERK inhibitor, PD98059, was used. As shown in Figure 6, the tyrosinase activity enhanced by α-MSH, α-MSH, and PD98059 cocktails was attenuated by PH treatment. The results confirmed that PH inhibits melanogenesis by attenuating MITF expression via the ERK and p38 signaling pathways in the cells.

### 2.5. Effects of PH on the AKT Signaling Pathway in B16F10 Cells

**Figure 7:**
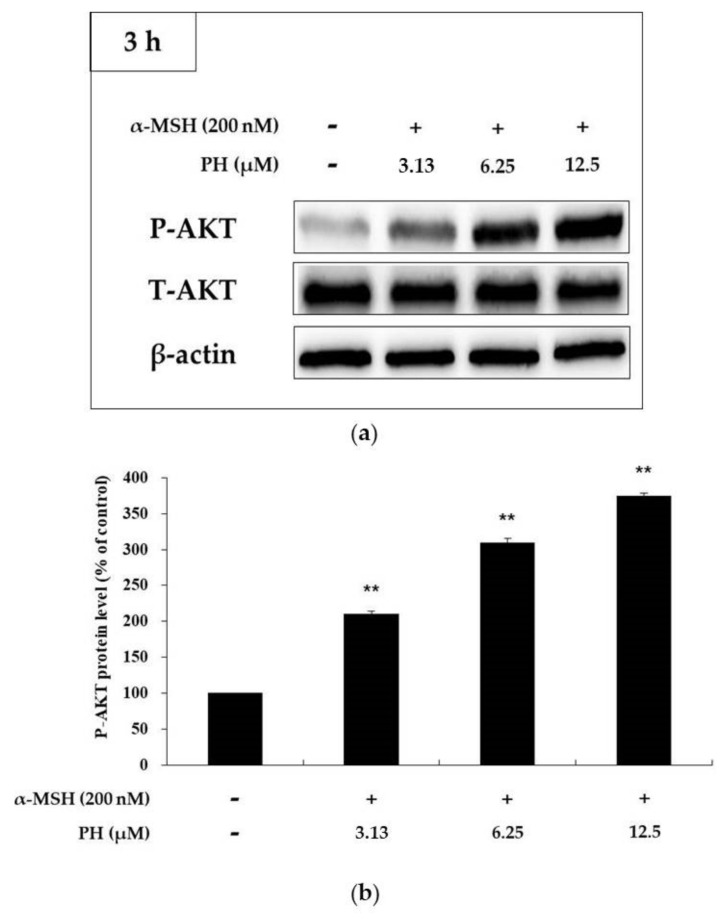
Effects of PH on AKT phosphorylation. B16F10 cells were treated with PH at the indicated concentrations for 3 h. (**a**) The Western blotting results and (**b**) protein levels of p-AKT. Results are expressed as percentages of the untreated cells. All experiments were carried out in triplicate, and the results are expressed as the mean ± SD. ** *p* < 0.01. P: phosphorylated, T: total.

Recent studies have reported that the increased phosphorylation of AKT attenuates melanogenesis in B16F10 cells [24,25]. Thus, to confirm that PH is related to the regulation of AKT phosphorylation, we treated α-MSH-stimulated cells with various concentrations of PH. The results showed that the phosphorylation of AKT was significantly increased in a concentration-dependent manner (Figure 7), suggesting that PH decreases melanogenesis by increasing AKT phosphorylation.

### 2.6. PH Inhibits Melanogenesis in Normal Human Epidermal Melanocytes

**Figure 8:**
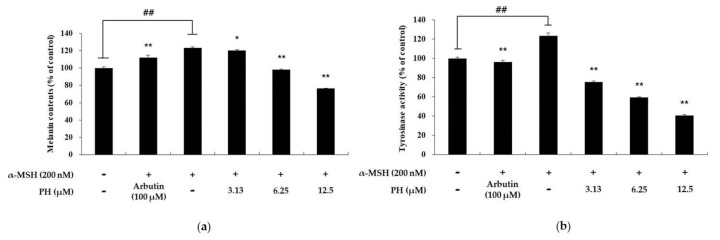
Effects of PH on melanin production and tyrosinase activity in normal human melanocytes. Cells were treated with PH at the indicated concentrations for 72 h. α-MSH (200 nM) and arbutin (100 μM) were used as a negative and positive control, respectively. (**a**) The melanin content and (**b**) tyrosinase activity are expressed as percentages relative to untreated cells. All experiments were carried out in triplicate, and the results are expressed as the mean ± SD. ^##^ *p* < 0.01, compared with the untreated cell group and * *p* < 0.05, ** *p* < 0.01, compared with the α-MSH-treated group.

To confirm that the hypopigmentation effects of PH in murine melanoma cells also affect normal human melanocytes, we examined the melanin content and tyrosinase activity in human epidermal melanocytes treated with PH. α-MSH (200 nM) and arbutin (100 μM) were used as a negative and positive control, respectively. As shown in Figure 8, the melanin content and tyrosinase activity of α-MSH-treated cells increased by 23% and 24%, respectively, relative to the untreated control cells. In contrast, in the arbutin-treated group, tyrosinase activity was decreased by 9% and 22%, respectively, compared with the α-MSH-treated group. The melanin content of 3.13, 6.25, and 12.5 µM PH-treated groups was decreased by 2%, 20%, and 38%, respectively, compared with the α-MSH-treated group. In addition, the tyrosinase activity of the 3.13, 6.25, and 12.5 µM PH-treated groups was decreased by 40%, 52%, and 68%, respectively, compared with the α-MSH-treated group.

## 3. Discussion

Various polyphenolic compounds with bioactive effects exist in natural products and have been studied extensively [39,40]. The bioactive effects of resveratrol, a polyphenolic compound, are already documented. However, resveratrol is unstable in the environment. As a result, many derivatives of resveratrol have been studied, including PH [32,33,34]. In this study, we focused on the anti-melanogenic effects of PH and performed a mechanistic study to elucidate the signaling pathways in B16F10 cells. As a result, the treatment of α-MSH-stimulated cells with PH was found to attenuate melanogenesis. To determine the effects of PH on melanogenesis, tests for melanin content and tyrosinase activity were performed. In addition, Western blotting assays were used to confirm the protein levels of melanogenic enzymes, such as tyrosinase and TRP-1 and -2, as well as the transcriptional factor MITF. Western blotting was used to determine the effects of PH on the phosphorylation of MAPK and AKT. Lastly, tests for determining melanin content and tyrosinase activity were also performed to evaluate the effects of PH on melanogenesis in normal human melanocytes.

To determine the concentration of PH at which there is no cytotoxicity, MTT assay was performed. The results indicated that at concentrations 1.57, 3.13, 6.25, and 12.5 µM, PH did not affect cell viability (Figure 1). In addition, the PH treatment of α-MSH-stimulated cells decreased tyrosinase activity and melanin content in a dose-dependent manner (Figure 2).

Several studies have previously shown that the expression of melanogenic enzymes (tyrosinase, TRP-1, and TRP-2) is transcriptionally regulated by MITF, resulting in a decrease in melanogenic enzyme expression and the inhibition of melanogenesis in B16F10 melanoma cells [13,14,15,16,17]. Our results showed a decrease in the expression of MITF, as well as melanogenic enzymes, after PH treatment in α-MSH-induced cells in a concentration-dependent manner (Figure 3 and Figure 4). These results demonstrated that PH has an effect on MITF inhibition, which results in decreased melanogenic enzyme expression and leads to the downregulation of melanogenesis.

The AKT signaling pathway has been proposed to be involved in melanogenesis in B16F10 cells. Increased AKT phosphorylation decreases the phosphorylation of CREB to inhibit MITF expression and prevent the binding of MITF to the tyrosinase promoter, resulting in anti-melanin production [24,25]. To evaluate whether PH affects the AKT signaling pathway, we examined the level of AKT phosphorylation using Western blotting assays. Our results showed that PH significantly increased the phosphorylation of AKT (Figure 7), indicating that PH attenuates MITF expression via the activation of AKT phosphorylation, which results in anti-melanogenesis.

Previous studies have reported that MAPK signaling pathways are involved in melanogenesis. In MAPK, p38 and JNK phosphorylated MITF, modulating its transcriptional activity in response to specific environmental stimuli. Therefore, the suppression of p38 and JNK phosphorylation leads to the inhibition of melanogenesis via decreased MITF expression [41]. In contrast, the activation of ERK phosphorylation suppresses melanogenesis [21,22,23]. Previous reports have demonstrated that the phosphorylation of ERK induces MITF degradation via the ubiquitin-dependent proteasome pathway by phosphorylating serine 73 of MITF, which results in the inhibition of tyrosinase transcription [15,42]. As shown in Figure 5, PH significantly increased the phosphorylation of ERK and decreased p38 in a concentration-dependent manner. However, PH did not affect JNK phosphorylation. Thus, to confirm whether the ERK pathway is involved in melanogenesis, PD98059, an ERK inhibitor, was used. As shown in Figure 6, the tyrosinase activity enhanced by α-MSH, α-MSH, and PD98059 cocktails was attenuated by PH treatment. In addition, tyrosinase activity was inhibited by PH, but due to the ERK phosphorylation inhibitory action of PD98059, tyrosinase activity was less inhibited in the α-MSH, PD98059 and PH treated group compared to the α-MSH and PH treated group. This suggested that ERK phosphorylation is related to tyrosinase activity. Therefore, results demonstrated that PH inhibits melanogenesis by attenuating MITF expression via the ERK and p38 signaling pathways in the cells. Additionally, to confirm that the anti-melanogenic effect of PH in murine melanoma cells also affects normal human melanocytes, we measured the melanin content and tyrosinase activity in human epidermal melanocytes treated with PH. As a result, tyrosinase activity and melanin contents were found to be inhibited by PH treatment (Figure 8), indicating that PH may also affect anti-melanogenesis in normal human melanocytes.

In summary, this study is the first to evaluate the anti-melanogenic effect of PH, which was found to decrease melanogenesis in B16F10 cells. Additionally, our data showed that PH increased ERK and AKT phosphorylation and decreased p38 phosphorylation in MAPK/AKT signaling pathways, which resulted in MITF suppression, leading to anti-melanogenic effects. We also found that the tyrosinase activity and melanin content were inhibited by PH treatment in B16F10 melanoma and normal human melanocytes cells. The data reveal that PH attenuates melanogenesis by activating ERK and AKT and inhibiting p38 phosphorylation in MAPK/AKT signaling pathways. Therefore, the results suggested that PH has potential for use in the treatment of hyperpigmentation disorders. Additionally, PH could be used as a component in skin-whitening products. However, further studies will be needed in order to evaluate the safety and efficacy of PH.

## 4. Materials and Methods

### 4.1. Chemicals and Reagents

Pinostilbene hydrate, dimethyl sulfoxide (DMSO), α-MSH, NaOH, 3-(4,5-dimethylthiazol-2-yl)-2,5-diphenyltetrazolium bromide (MTT), radioimmunoprecipitation assay (RIPA) buffer, protease inhibitor cocktail, and L-DOPA were purchased from Sigma-Aldrich (St. Louis, MO, USA). Dulbecco’s modified Eagle’s medium (DMEM), fetal bovine serum (FBS), penicillin/streptomycin (P/S), trypsin–ethylenediaminetetraacetic acid (T/E), bicinchoninic acid assay (BCA) kit, and PD98059, an ERK inhibitor, were obtained from Thermo Fisher Scientific (Waltham, MA, USA). Antibodies against tyrosinase, TRP-1, TRP-2, and MITF were purchased from Santa Cruz Biotechnology (Dallas, TX, USA). Antibodies against phospho-p38, p38, phospho-JNK, JNK, phospho-ERK, ERK, phospho-AKT, AKT, and β-actin were obtained from Cell Signaling Technology (Danvers, MA, USA). An enhanced chemiluminescence (ECL) kit, phosphate-buffered saline (PBS), radioimmunoprecipitation assay (RIPA) buffer, and 2× Laemmli sample buffer were obtained from Biosesang (Sungnam, Gyeonggi-do, Korea) and Bio-Rad (Hercules, CA, USA), respectively.

### 4.2. Cell Culture

Murine melanoma B16F10 melanoma cells were obtained from the Korean Cell Line Bank (Seoul, Korea) and cultured in phenol red-free DMEM supplemented with 10% FBS and 1% penicillin/streptomycin in a humidified atmosphere containing 5% CO_2_ at 37 °C and sub-cultured every 96 h. Cells were treated with PH for 72 h (for tyrosinase activity and melanin content assay), and α-MSH (200 nM) and arbutin (100 μM) were used as negative and positive controls, respectively. Human epidermal melanocytes (moderately pigmented donor, HEMn-MP) were purchased from Life Technologies (Carlsbad, CA, USA). The cells were cultured in 254 medium (Life Technologies) supplemented with 1% human melanocyte growth supplements and 1% penicillin/streptomycin in 5% CO_2_ at 37 °C.

### 4.3. Measurement of Cell Viability

Cell viability was measured using the MTT assay. Cells were cultured in 24-well plates for 24 h and then treated with 1.57, 3.13, 6.25, and 12.5 µM PH for 72 h. The MTT solution was added to the wells and removed after incubation for 4 h. After washing twice with phosphate-buffered saline (PBS), the formazan crystals formed were dissolved in 1 mL of DMSO. The absorbance was measured at 540 nm using an ELISA reader.

### 4.4. Measurement of Intracellular Melanin Content

Cells (5.0 × 10^4^ cells/well) were seeded in 6-well plates for 24 h and then treated with 1.57, 3.13, 6.25, and 12.5 µM PH and α-MSH (200 nM), followed by incubation at 37 °C with 5% CO_2_ for 72 h. After removing the cultured media, the cells were washed twice with PBS and lysed in 1 mL of 1 N NaOH for 1 h at 80 °C. The experiment was carried out in triplicate, and the released melanin was measured using an ELISA reader at 405 nm.

### 4.5. Measurement of Intracellular Tyrosinase Activity

Cells (8.0 × 10^4^ cells/dish) were seeded in 60-mm dishes and incubated for 24 h in the culture medium. Next, the cells were treated with 3.13, 6.25, and 12.5 µM PH, α-MSH (200 nM), and arbutin (100 μM), followed by incubation for 72 h at 37 °C and 5% CO_2_ under humidified conditions. After incubation, the cultured media were removed, and the cells were collected into e-tubes and lysed in RIPA buffer containing a 1% protease inhibitor cocktail. Cell lysates were vortexed every 10 min for 30 min and centrifuged for 25 min at 15,000× *g.* The supernatant was collected, and the protein level was adjusted using a BCA kit. Next, 20 μL of each adjusted protein sample and 80 μL of L-DOPA (2 mg/mL) were mixed in the well of a 96-well plate to perform the tyrosinase activity test. After incubation at 37 °C for 2 h, absorbance was measured at 490 nm using an ELISA reader.

### 4.6. Western Blot Analysis

Cells (8.0 × 10^4^ cells/dish) were seeded into 60-mm dishes and incubated for 24 h in the culture medium. Next, the cells were treated with 3.13, 6.25, and 12.5 µM PH, α-MSH (200 nM), and arbutin (100 μM), followed by incubation for 1, 2, 3, 4, and 24 h at 37 °C and 5% CO_2_. After collection, the cells were lysed in RIPA buffer containing a 1% protease inhibitor cocktail. The cell lysates were vortexed every 10 min for 30 min and centrifuged for 25 min at 15,000 rpm. The supernatant was collected, and the protein level was adjusted using a BCA kit. The adjusted lysate and 2× Laemmli sample buffer were mixed in an e-tube and heated for 5 min to prepare the sample for Western blotting. Samples containing equal amounts of protein (in 20 μL) were loaded on 10% sodium dodecyl sulfate-polyacrylamide gels for 1 h at 150 V. The separated proteins were then transferred to a polyvinylidene difluoride membrane, which was blocked with 1× TBST containing 5% skim milk for 1 h, and then washed six times with Tris-buffered saline (20 mM Tris base, 137 mM NaCl, pH 7.6) containing 0.1% Tween-20 (TBST). The membrane was incubated overnight at 4 °C with primary antibodies diluted in TBST (1:1000), washed six times with TBST, and incubated with secondary antibodies diluted in TBST (1:3000) for 1 h, followed by washing three times with TBST. The target protein bands were detected using an ECL kit.

### 4.7. Data Analysis

All experiments were carried out in triplicate, and the results are expressed as the mean ± SD. Differences between the control and treatment groups were evaluated by one-way analysis of variance (ANOVA) using SPSS (v. 22.0, SPSS Inc., Chicago, IL, USA). The significance value was determined as *p* < 0.05 (*), *p* < 0.01 (**), *p* < 0.01 (aa), *p* < 0.01 (bb).

## References

[1] Hearing V.J. (2005). Biogenesis of pigment granules: A sensitive way to regulate melanocyte function. J. Dermatol. Sci..

[2] Bonaventure J., Domingues M.J., Larue L. (2013). Cellular and molecular mechanisms controlling the migration of melanocytes and melanoma cells. Pigment Cell Melanoma Res..

[3] Tadokoro T., Yamaguchi Y., Batzer J., Coelho S.G., Zmudzka B.Z., Miller S.A., Wolber R., Beer J.Z., Hearing V.J. (2005). Mechanisms of skin tanning in different racial/ethnic groups in response to ultraviolet radiation. J. Invest. Dermatol..

[4] Tabassum N., Hamdani M. (2014). Plants used to treat skin diseases. Pharmacogn. Rev..

[5] D’ISCHIA M., Wakamatsu K., Cicoira F., Mauro E.D., Garcia-Borron J.C., Commo S., Galván I., Ghanem G., Kenzo K., Meredith P. (2015). Melanins and melanogenesis: From pigment cells to human health and technological applications. Pigment Cell Melanoma Res..

[6] Brenner M., Hearing V.J. (2008). The protective role of melanin against UV damage in human skin. Photochem. Photobiol..

[7] Miyamura Y., Coelho S.G., Wolber R., Miller S.A., Wakamatsu K., Zmudzka B.Z., Ito S., Smuda C., Passeron T., Choi W. (2007). Regulation of human skin pigmentation and responses to ultraviolet radiation. Pigment Cell Res..

[8] Briganti S., Camera E., Picardo M. (2003). Chemical and instrumental approaches to treat hyperpigmentation. Pigment Cell Res..

[9] Sugumaran M. (2016). Reactivities of quinone methides versus o-quinones in catecholamine metabolism and eumelanin biosynthesis. Int. J. Mol. Sci..

[10] Hearing V.J., Jimenez M. (1987). Mammalian tyrosinase—The critical regulatory control point in melanocyte pigmentation. Int. J. Biochem..

[11] Jiménez-Cervantes C., Solano F., Kobayashi T., Urabe K., Hearing V.J., Lozano J.A., Garcia-Borrón J.C. (1994). A new enzymatic function in the melanogenic pathway. The 5,6-dihydroxyindole-2-carboxylic acid oxidase activity of tyrosinase-related protein-1 (TRP1). J. Biol. Chem..

[12] Tsukamoto K., Jackson I.J., Urabe K., Montague P.M., Hearing V.J. (1992). A second tyrosinase-related protein, TRP-2, is a melanogenic enzyme termed DOPAchrome tautomerase. EMBO J..

[13] Sun M., Xie H.F., Tang Y., Lin S.Q., Li J.M., Sun S.M., Hu X.L., Huang Y.X., Shi W., Jian D. (2017). G protein-coupled estrogen receptor enhances melanogenesis via cAMP-protein kinase (PKA) by upregulating microphthalmia-related transcription factor-tyrosinase in melanoma. J. SteroidBiochem. Mol. Biol..

[14] Bentley N.J., Eisen T., Goding C.R. (1994). Melanocyte-specific expression of the human tyrosinase promoter: Activation by the microphthalmia gene product and role of the initiator. Mol. Cell. Biol..

[15] Vachtenheim J., Borovansky J. (2010). “Transcription physiology” of pigment formation in melanocytes: Centralrole of MITF. Exp. Dermatol..

[16] Chan C.F., Huang C.C., Lee M.Y., Lin Y.S. (2014). Fermented broth in tyrosinase- and melanogenesis inhibition. Molecules.

[17] Kim A., Yim N.H., Im M., Jung Y.P., Liang C., Cho W.K., Ma J.Y. (2013). Ssanghwa-tang, an oriental herbal cocktail, exerts anti-melanogenic activity by suppression of the p38 MAPK and PKA signaling pathways in B16F10 cells. BMC Complement. Altern. Med..

[18] Ahn J.H., Jin S.H., Kang H.Y. (2008). LPS induces melanogenesis through p38 MAPK activation in human melanocytes. Arch. Dermatol. Res..

[19] Widlude H.R., Fisher D.E. (2003). Microphthalmia-associated transcription factor: A critical regulator of pigment cell development and survival. Oncogene.

[20] Saha B., Singh S.K., Sarkar C., Bera R., Ratha J., Tobin D.J., Bhadra R. (2006). Activation of the Mitf promoter by lipid-stimulated activation of p38-stress signalling to CREB. Pigment Cell Res..

[21] Wu L.C., Lin Y.Y., Yang S.Y., Weng Y.T., Tsai Y.T. (2011). Antimelanogenic effect of c-phycocyanin through modulation of tyrosinase expression by upregulation of ERK and downregulation of p38 MAPK signaling pathways. J. Biomed. Sci..

[22] Kang S.J., Choi B.R., Lee E.K., Kim S.H., Yi H.Y., Park H.R., Song C.H., Lee Y.J., Ku S.K. (2015). Inhibitory Effect of Dried Pomegranate Concentration Powder on Melanogenesis in B16F10 Melanoma Cells; Involvement of p38 and PKA Signaling Pathways. Int. J. Mol. Sci..

[23] Han J.S., Sung J.H., Lee S.K. (2016). Antimelanogenesis Activity of Hydrolyzed Ginseng Extract (GINST) via Inhibition of JNK Mitogen-activated Protein Kinase in B16F10 Cells. J. Food Sci..

[24] Im D.S., Lee J.M., Lee J., Shin H.J., No K.T., Park S.H., Kim K. (2017). Inhibition of collagenase and melanogenesis by ethanol extracts of *Orostachys japonicus A. Berger*: Possible involvement of Erk and Akt signaling pathways in melanoma cells. Acta Biochim. Biophys. Sin..

[25] Yao C., Jin C.L., Oh J.H., Oh I.G., Park C.H., Chung J.H. (2015). *Ardisia crenata* extract stimulates melanogenesis in B16F10 melanoma cells through inhibition ERK1/2 and Akt activation. Mol. Med. Rep..

[26] Shrotriya S., Agarwal R., Sclafani R.A. (2015). A perspective on chemoprevention by resveratrol in head and neck squamous cell carcinoma. Adv. Exp. Med. Biol..

[27] Varoni E.M., Lo Faro A.F., Sharifi-Rad J., Iriti M. (2016). Anticancer molecular mechanisms of resveratrol. Front. Nutr..

[28] Buhrmann C., Shayan P., Kraehe P., Popper B., Goel A., Shakibaei M. (2015). Resveratrol induces chemosensitization to 5-fluorouracil through upregulation of intercellular junctions, Epithelial-to-mesenchymal transition and apoptosis in colorectal cancer. Biochem. Pharmacol..

[29] Ma L., Li W., Wang R., Nan Y., Wang Q., Liu W., Jun F. (2015). Resveratrol enhanced anticancer effects of cisplatin on non-small cell lung cancer cell lines by including mitochondrial dysfunction and cell apoptosis. Int. J. Oncol..

[30] Fremont L. (2000). Biological effects of resveratrol. Life Sci..

[31] Lin F.Y., Hsieh Y.H., Yang S.F., Chen C.T., Tang C.H., Chou M.Y., Chuang Y.T., Lin C.W., Chen M.K. (2015). Resveratrol suppresses TPA-induced matrix metalloproteinase-9 expression through the inhibition of MAPK pathways in oral cancer cells. J. Oral. Pathol. Med..

[32] Wen W., Lowe G., Roberts C.M., Finlay J., Han E.S., Glackin C.A., Dellinger T.H. (2018). Pterostilbene Suppresses Ovarian Cancer Growth via Induction of Apoptosis and Blockade of Cell Cycle Progression Involving Inhibition of the STAT3 Pathway. Int. J. Mol. Sci..

[33] Mikstacka R., Przybylska D., Rimando A.M., Baer-Dubowska W. (2007). Inhibition of human recombinant cytochromes P450 CYP1A1 and CYP1B1 by trans-resveratrol methyl ethers. Mol. Nutr. Food Res..

[34] Chao J., Li H., Cheng K.W., Yu M.S., Chang R.C., Wang M. (2010). Protective effects of pinostilbene, a resveratrol methylated derivative, against 6-hydroxydopamine-induced neurotoxicity in SH-SY5Y cells. J. Nutr. Biochem..

[35] Sun Y., Wu X., Cai X., Song M., Zheng J., Pan C., Qiu P., Zhang L., Zhou S., Tang Z. (2016). Identification of pinostilbene as a major colonic metabolite of pterostilbene and its inhibitory effects on colon cancer cells. Mol. Nutr. Food Res..

[36] Hsieh M.J., Chin M.C., Lin C.C., His Y.T., Lo Y.S., Chuang Y.C., Chen M.-K. (2018). Pinostilbene hydrate suppresses human oral cancer cell metastasis by downregulation of matrix metalloproteinase-2 through the mitogen-activated protein kinase signaling pathway. Cell Physiol. Biochem..

[37] Wang T.T., Schoene N.W., Kim Y.S., Mizuno C.S., Rimando A.M. (2010). Differential effects of resveratrol and its naturally occurring methylether analogs on cell cycle and apoptosis in human androgen-responsive LNCaP cancer cells. Mol. Nutr. Food Res..

[38] Uesugi D., Hamada H., Shimoda K., Kubota N., Ozaki S., Nagatani N. (2017). Synthesis, Oxygen Radical Absorbance Capacity, and Tyrosinase Inhibitory Activity of Glycosides of Resveratrol, Pterostilbene, and Pinostilbene. Biosci. Biotechnol. Biochem..

[39] Nathan R.P., Julia L.B. (2009). A Review of the Antioxidant Mechanisms of Polyphenol Compounds Related to Iron Binding. Cell Biochem. Biophys..

[40] Yemmen M., Landolsi A., Hamida J.B., Mégraud F., Trabelsi Ayadi M. (2017). Antioxidant activities, anticancer activity and polyphenolics profile, of leaf, fruit and stem extracts of *Pistacia lentiscus* from Tunisia. Cell. Mol. Biol..

[41] D’Mello S.A.N., Finlay G.J., Baguley B.C., Askarian-Amiri M.E. (2016). Signaling Pathways in Melanogenesis. Int. J. Mol. Sci..

[42] Kim D.S., Kim S.Y., Chung J.H., Kim K.H., Eun H.C., Park K.C. (2002). Delayed ERK activation by ceramide reduces melanin synthesis in human melanocytes. Cell Signal..

